# Gastrointestinal Polyparasitism in Bushmeat in Zadie Department in Northeast Gabon

**DOI:** 10.3390/vetsci10030229

**Published:** 2023-03-17

**Authors:** Gael Darren Maganga, Patrice Makouloutou-Nzassi, Larson Boundenga, Hurlis Nesla Maganga Landjekpo, Felicien Bangueboussa, Telstar Ndong Mebaley, Franck Mounioko, Oubri Bassa Gbati

**Affiliations:** 1 Unité Emergence des Maladies Virales, Centre Interdisciplinaire de Recherches Médicales de Franceville (CIRMF), Franceville BP 769, Gabon; gael_maganga@yahoo.fr (G.D.M.); landjekpohurlis@gmail.com (H.N.M.L.); telstarlunique@gmail.com (T.N.M.); 2 Institut National Supérieur d’Agronomie et de Biotechnologie (INSAB), Université des Sciences et Techniques de Masuku (USTM), Franceville BP 913, Gabon; 3 Département de Biologie et Ecologie Animale, Institut de Recherche en Ecologie Tropicale (IRET/CENAREST), Libreville BP 13354, Gabon; 4 Unité de Recherche en Ecologie de la Santé, Centre Interdisciplinaire de Recherches Médicales de Franceville (CIRMF), Franceville BP 769, Gabon; boundenga@gmail.com (L.B.); bangueboussafelicien040@gmail.com (F.B.); fmounioko@yahoo.fr (F.M.); 5 Department of Anthropology, Durham University, South Road, Durham DH1 3LE, UK; 6 Biology Department, Masuku University of Sciences and Technic (USTM), Franceville BP 943, Gabon; 7 Département de Santé Publique-Environnement, Ecole Inter-Etats des Sciences et Médecine Vétérinaires (EISMV), Dakar BP 5077, Senegal; oubribassa@yahoo.fr

**Keywords:** wild animal, bushmeat, gastrointestinal parasites, prevalence, Zadie department, Gabon

## Abstract

**Simple Summary:**

Wildlife is an important source of infectious pathogens, including parasites. Intestinal parasites are among the parasites associated with outbreaks of foodborne disease. This article analyses gastrointestinal parasites in fecal and intestine samples from wild animals used as bushmeat in the Zadie Department, Gabon. Identified parasites belonged to Fifteen taxa of gastrointestinal parasites, some of which are pathogenic for the human being. Gastrointestinal parasite detected in fecal samples from wildlife poses risks to humans, animal, and agricultural production due to the possibility of direct contact with feces. Much care should be given when manipulating games, particularly offal. In conclusion, monitoring wildlife parasites should be conducted in the One Health approach, which recognizes the close link between human, animal, plant, and ecosystem health.

**Abstract:**

Wild animals harbor pathogens that can be infectious agents for humans, including parasites. This study aimed to identify gastrointestinal parasites and assess their prevalence and the potential risk for humans associated with consuming these animals. The research was conducted from August to December 2019. Parasitological analyses were carried out on the feces and intestines of 113 wild animals, including antelopes (24), duikers (58), porcupines (18), small monkeys (*Cercopithecus*) (8), nandinia (2), pangolin (1), genet (1), and a crocodile (1), from the Zadié Department in the province of Ogooué-Ivindo in the northeast of Gabon. The results revealed 15 taxa of gastrointestinal parasites, including nine nematodes: *Strongylids* (61/113), *Strongyloides* spp. (21/113), *Ascaris* spp. (21/113), *Trichuris* spp. (39/113), *Capillaria* spp. (9/113), *Protostrongylus* spp. (5/113), *Enterobius* spp. (8/113), *Toxocara* spp. (7/113) and *Mammomonogamus* spp. (5/113); three species of protozoa, namely *Balantidium* spp. (12/113), *Eimeria* spp. (17/113), and *Entamoeba* spp. (9/113); two species of trematodes, namely *Fasciola* spp. (18/113) and *Paramphistomum* spp. (21/113); and cestode species, *Taenia* spp. (1/113). The prevalence of gastrointestinal parasitism in these animals was 85.84% (97/113). In addition, among these parasitic taxa, some are potential pathogens for humans, such as *Ascaris* spp., *Balantidium* spp., *Entamoeba* spp., and *Taenia* spp. The consumption of games, particularly offal, infested by these parasites, could threaten human health.

## 1. Introduction

For humans, wildlife represents a source of varying interest in economic, cultural, tourist, scientific, and food terms. Regarding this last point, the meat of wild animals, called “bushmeat” by African populations [1], is a significant source of protein for hundreds of millions of people around the world [2]. Bushmeat consumption provides a large share of animal protein for many rural families in Central Africa [2,3]. Indeed, it represents nearly 30 to 80% of the protein intake for the populations bordering the forests of the Congo Basin [4,5]. In Gabon particularly, in 2005, the annual bushmeat consumption had already been estimated at 15,000 tons per year [6], making this country one of the biggest bushmeat consumers. In Gabon, particularly in the Ogooué-Ivindo province, the bushmeat trade has become the primary source of income for 47% of households. Indeed, it is one of the provinces of Gabon where hunting is crucial for family consumption or sale to meet protein and financial needs. Nearly 20% of hunters from surrounding villages regularly sell bushmeat in town [7].

Furthermore, since the 1940s, there has been an increase in the incidence of infectious diseases. Zoonotic pathogens cause the majority (60.3%) of these infectious diseases. In addition, an estimated 71.8% of zoonotic infectious diseases are caused by pathogens from wildlife. Among these pathogens are protozoa 10.7% and helminths 3.3% [8]. Thus, parasites can be zoonotic and involve vertebrate animals. These animals, therefore, constitute reservoirs of parasites and, for some, the direct source of human contamination following their ingestion as food [9,10]. If the consumption of games proves dangerous for humans, the parasitism associated with these animals could also threaten their conservation. Indeed, parasitic infection and its complications are significant threats to wild animal populations and can act as an agent of population declines or species extinction [11,12]. Although it appears that wildlife has adapted to the presence of parasites, it has not adapted to the adverse effects of parasitism [13,14].

Thus, this study sought to determine the prevalence of gastrointestinal parasites in wild animals from the Department of Zadié in the province of Ogooué-Ivindo, Gabon, and investigate other parameters associated with its presence, e.g., examine its distribution according to species, sex, and age.

## 2. Materials and Methods

### 2.1. Study Areas and Sample Collection

**Figure 1:**
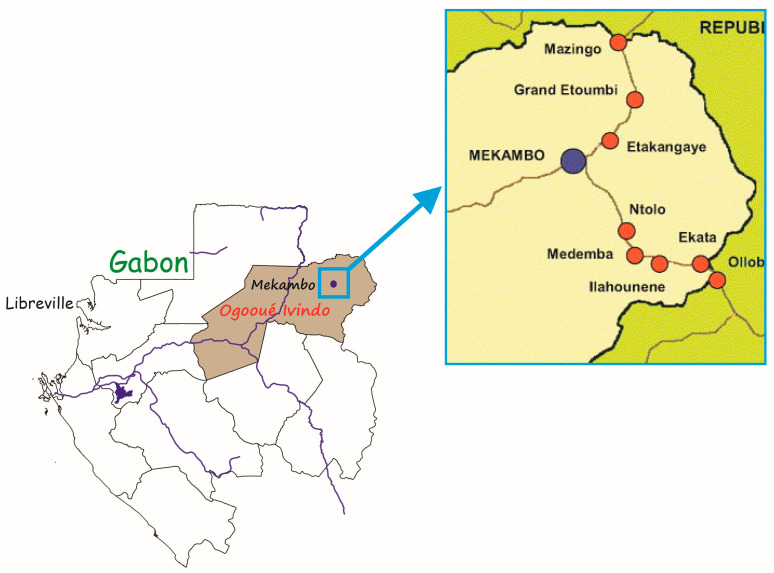
The sampling villages (represented by red dot) of bushmeat in the Zadie department.

The study was conducted in 11 villages in the department of Zadié, province of Ogooué-Ivindo, in northeast Gabon (Figure 1). The sampling occurred in April, June, and July of 2019. Fecal and intestinal samples were collected post-mortem from 113 bushmeat of different species, including antelope, duiker, porcupine, *Cercopithecus*, small pangolin, nandinia, genet, and crocodiles (Table 1). The feces were collected directly from the rectum or after dissection and incision of the cecum using disposable hand gloves to prevent contamination and ensure maximum protection. When the sample was obtained, it was immediately fixed in 10% neutral buffered formalin in a 50 mL falcon tube and labeled correctly. All the samples were exported to the CIRMF Parasitology laboratory unit for analysis. For each sample, we recorded species, individual ID, sex, age class, and the collection place.

### 2.2. Parasitological Analysis

All the fecal samples were microscopically screened for helminth eggs and larvae. The isolation of parasitic larvae, eggs, and cysts underwent treatment using two techniques, flotation, and sedimentation, as per Dryden et al. [15] and Gillespie [16]. In the flotation step, we applied a fecal straining procedure in which 2 g of fecal sample were (a) diluted in 14 mL of saturated salt solution (40% of NaCl), (b) strained of large debris through a sieve, and two layers of compress, (c) transferred to and filled a 15 mL falcon until a slightly positive meniscus was formed, (d) had a glass coverslip placed over it gently and incubated for 10 min, (e) had the coverslip carefully removed and placed onto a clean slide for observation. We followed the centrifugal protocol outlined by [16] for concentration procedures with the fecal sample or the fecal pellet remaining after the previously described flotation methodology. Irrespective of the technique, the slides were fully andthoroughly evaluated for parasitic forms at 40× and 100× using an optical microscope equipped with a camera (Leica, Microsystems). Morphological features such as shape, size, and color were used for identification [17]. In addition to these techniques, each collected intestinal material was dissected, scratched, washed with tap water, and carefully observed for helminths under the dissection microscope. The isolated parasites were identified according to standard morphological characteristics at the genus level [17], and images of the representative parasites were taken.

### 2.3. Statistical Analysis

The statistical analyses were performed using R software [18]. The parasite infestation rates were calculated as the proportion of the positive results among the total number tested, and they were given as percentages. The parasite infestation rates based on the bushmeat species, sex, and age were calculated and compared using the Chi-square (χ^2^) test. The difference with a *p*-value of less than 0.05 (*p* < 0.05; 95% confidence interval) was considered for statistical significance.

## 3. Results

### 3.1. Types of Parasites Taxa Identified

**Figure 2:**
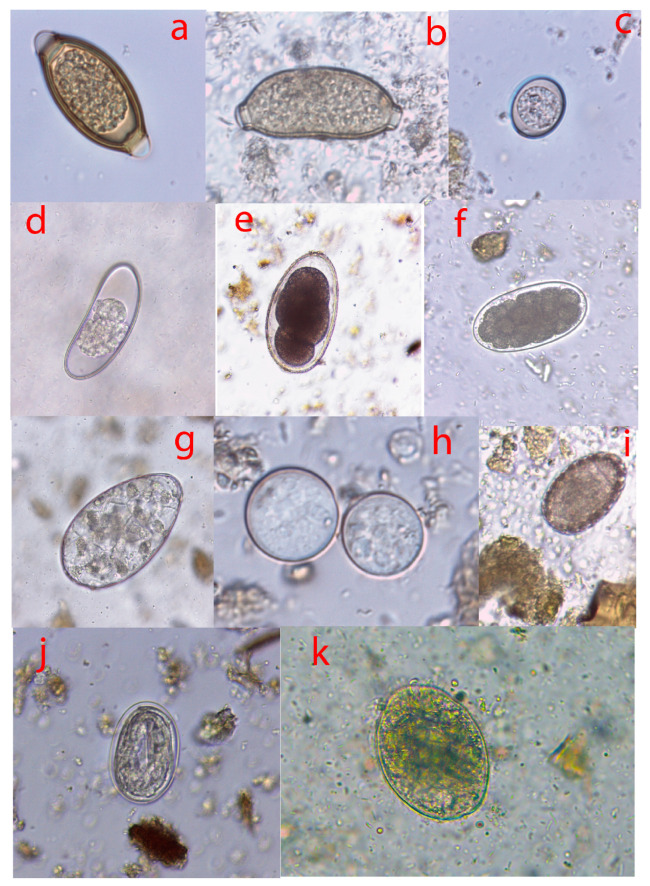
Micrographs of gastrointestinal parasite eggs isolated from different bushmeat in the Zadie department. (**a**) *Trichuris* spp.; (**b**) *Capillaria* spp.; (**c**) *Eimeria* spp.; (**d**) *Enterobius* spp.; (**e**) *Mammomonogamus* spp.; (**f**) *Strongylid* egg; (**g**) *Fasciola* spp.; (**h**) *Entamoeba* spp.; (**i**) *Ascaris* spp.; (**j**) *Strongyloïdes* spp.; (**k**) *Balantidium* spp.

**Figure 3:**
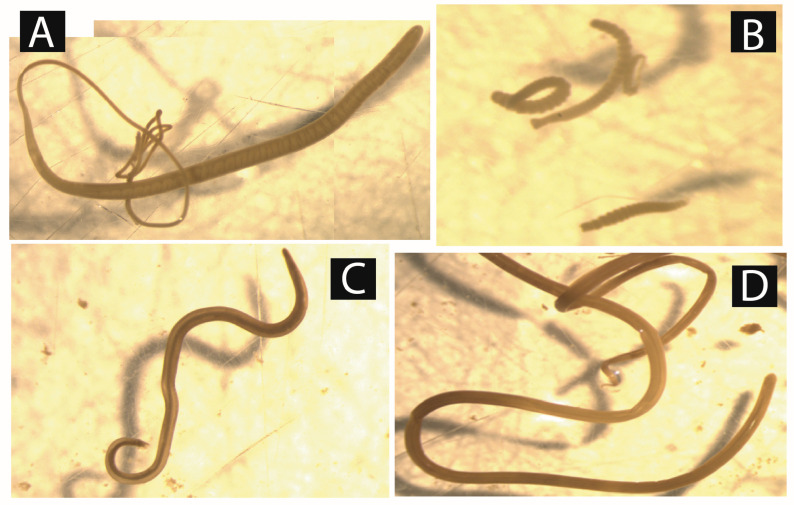
Micrographs of gastrointestinal parasite specimens isolated from different bushmeat in the Zadie department. (**A**) *Trichuris* spp.; (**B**) *Taenia* spp.; (**C**) *Toxocara* spp.; (**D**) *Ascaris* spp.

After coprological analysis, 13 parasites were recovered from the studied animals. They included *Strongylids* species, *Ascaris* spp., *Balantidium* spp., *Capillaria* spp., *Eimeria* spp., *Entamoeba* spp., *Enterobius* spp., *Fasciola* spp., *Mammomonogamus* spp., *Paramphistomum* spp., *Protostrongylus* sp., *Strongyloïdes* spp., and *Trichuris* spp. (Figure 2). The intestinal content screening confirmed bushmeat infestation by *Ascaris* spp., *Trichuris* spp., *Strongyloïdes* spp., and *Strongylids* species. However, *Toxocara* spp. and *Taenia* spp. have also been identified (Figure 3). All these parasites were distributed differently between sex and age.

### 3.2. Overall Parasite Prevalence and Infestation Rate of Identified Parasites

Out of all examined bushmeat, the overall prevalence of parasitic infection was 85.8% (97/113). Furthermore, *Strongylids* eggs were more predominant, with an overall prevalence of 54% (61/113), followed by *Trichuris* spp. (34.5%, i.e., 39/113), *Ascaris* spp. (18.6%, or 21/113) and *Paramphistomum* spp. (18.6%, or 21/113) (Table 2). The least prevalent parasite was *Taenia* spp., with an infection rate of 0.9%. Five taxa were detected in the intestine as an adult and as eggs, simultaneously, and the remaining as eggs (7) or as a cyst (3) (Table 2). However, isolation in two different parasitic forms has a little incidence on the prevalence rate (Table 3).

### 3.3. Factors Influencing Gastrointestinal Parasitism in Bushmeat

#### 3.3.1. The Type of Bushmeat

The overall parasite infestation rate was higher in *Cercopithecus* (small monkeys) (100%, or 8/8), followed by antelopes (91.6%, 22/24), porcupines (83.3%, 15/18), and duikers (82.4%, 49/57) (Table 3). Overall, parasite diversity was larger in duikers, antelopes, and porcupines, with 13, 12, and 11 of the 15 parasite genera identified. *Strongylids* species were present in antelopes, duikers, genets, pangolin, *Cercopithecus* (small monkeys), and porcupines, i.e., in six of the eight animal species studied. Moreover, *Fasciola* spp. and *Paramphistomum* spp. were only present in two species, antelopes and duikers. *Taenia* spp. was detected only in a single genet. The *Strongylids* group was the most prevalent parasitic genera (61/97), followed by *Trichuris* spp. (38/97), which were present in antelopes, duikers, porcupines, and monkeys. At the same time, the least prevalent were *Taenia* spp. (1/97), present in genet only.

In addition, our results also highlighted the presence of coccidia of the genus *Eimeria* spp. in antelopes (4.4%), duikers (8.8%), and porcupines (1.8%) and revealed infestation of antelopes and duikers by the trematodes *Fasciola* spp. (8.0% and 7.9%, respectively) and *Paramphistomum* spp. (5.3% and 13.3%).

#### 3.3.2. Sex

Considering the sex, male animals had a prevalence rate of 88.7% (47/53) while female animals were infested at 83.05% (49/59); however, the difference was not significant (*p* > 0.05). The males harbored 14 parasite species, whereas the females harbored 13. Uniquely, *Toxocara* spp. and *Taenia* spp. were isolated in male animals, whereas, *Protostrongylus* spp. was isolated in females. For *Strongylids* species (54.7% vs. 52.5%), *Eimeria* spp. (18.9% vs. 11.9%) and *Paramphistomum* spp. (19.0% vs. 5.1%), the rate was higher in males than in females. However, the data were significant only for *Paramphistomum* spp. (*p* < 0.05). For *Strongyloides* spp. (23.7% against 13.2%), *Fasciola* spp. (18.6% vs. 17.0%), *Ascaris* spp. (22.0% against 15.1%), *Balantidium* spp. (11.9% against 9.4%), *Capillaria* spp. (11.9% versus 3.8%), *Entamoeba* spp. (10.2% vs. 5.7%), *Enterobius* spp. (8.5% vs. 3.8%) and *Mammomonogamus* spp. (5.1% versus 3.8%), the prevalence rates were higher in females than in males; however, the differences were insignificant (*p* > 0.05) (Table 4). On the contrary, *Trichuris* spp. seems to equally infect both sexes without significance (*p* > 0.05).

#### 3.3.3. Age

In the current study, regarding the infestation rate of total gastrointestinal parasites, adult animals had an infection rate of 85.4% while young animals had that of 88.2%; however, the data were insignificant (*p* > 0.05). The adults also harbored a more diverse parasite species than the young (15 vs. 12). Interestingly, *Strongyloides* spp., *Trichuris* spp., and *Enterobius* spp. were dominant in the adults. In contrast, *Strongylids* species, *Ascaris* spp., *Protostrongylus* spp., *Capillaria* spp., *Toxocara* spp., *Eimeria* spp., *Balantidium* spp., *Fasciola* spp. and *Paramphistomum* spp. were dominant in young animals, with the difference being significant only for *Protostrongylus* spp. (*p* = 0.025). Uniquely, *Entamoeba* spp., *Mammomonogamus* spp., and *Taenia* spp. were detected in the adults (Table 5).

## 4. Discussion

In this study, 113 samples collected from eight wild animal species were screened for the presence and diversity of gastrointestinal parasites. The prevalence rate (85.84%; *n* = 97) of GI parasites in bushmeat is lower than that reported by Okoye et al. (2015) [19] in Nsukka, Nigeria, namely 98.6%. These discrepancies might result from the total number of animals assayed (143 vs. 113) and the species composition of the samples.

In the current study, 15 parasites taxa were identified, namely *Balantidium* spp., *Toxocara* spp., *Taenia* spp., *Strongylids* species, *Eimeria* spp., *Entamoeba* spp., *Strongyloïdes* spp., *Ascaris* spp., *Trichuris* spp., *Capillaria* spp., *Protostrongylus* spp., *Enterobius* spp., *Fasciola* spp., *Paramphistomum* spp., and *Mammomonogamus* spp. These results demonstrate the vast diversity of gastrointestinal parasites found in wild animals. This diversity was reported by [19] in wild animals in Nigeria. In his study, 19 parasitic genera were detected, including eight of the genera identified in this study (*Strongylids*, *Eimeria* spp., *Entamoeba* spp., *Strongyloides* spp., *Ascaris* spp., *Trichuris* spp., *Capillaria* spp., *Enterobius* spp.). Furthermore, in another study carried out in Burkina Faso, Beleme and Bakoné [20] reported the presence of 12 taxa of gastrointestinal parasites at the Nazinga game ranch, of which three were detected in the present study (*Strongylids*, *Trichuris* spp., and *Paramphistomum* spp.) [20]. The authors screened 31 animals, including antelopes and buffaloes. However, in their study, these authors identified the parasites using a binocular magnifying glass after helminthological autopsies of the organs. Thus, the difference between their results and our study could be attributable to the techniques used. In addition, we searched for parasites using three methods (flotation, sedimentation, and microscopic observation of the intestinal content), which would have improved the detection rate of parasites compared to using a single method.

In the present work, *Strongylids* species was more predominant, with an overall prevalence of 54.0%, followed by *Trichuris* spp. (33.6%); however, in a study [19] based on fecal microscopic examination only, *Ascaris lumbricoides* was more predominant, with an overall prevalence of 48.8%, followed by *Trichuris trichiura* (23.1%), *Strongyloides papillosus* (21.7%) and *Trichostrongylus retortaeformis* (11.9%). On the other hand, *Oesophagostomum columbianum* had a lower prevalence of 7.7%. A study [20] conducted in Burkina Faso showed that *Trichostrongylus* sp. was present among the identified parasites but at a low rate, contrary to that obtained in our study (26.5%).

*Strongylids* spp. were present in antelopes at an infection rate of 75%, 41.4% in duikers, 100% in genet, 100% in pangolin, 50% in Cercopithecines (small monkeys), and 72.2% in porcupines. These results corroborate the work of several authors who have identified these parasites in antelopes, buffaloes, duikers, monkeys, and other wild mammals [19,20,21,22], confirming the fact that these parasite groups are herbivorous-mammalian gastrointestinal parasites [21,22]. *Toxocara* spp. was detected at a total infection rate of 6.2% in three of the eight examined animal species (antelopes, duiker, and *Cercopithecus*). This result follows that which was reported in [23], where the authors examined Walter’s Duiker collected from three bushmeat markets in Ondo State, Nigeria, for gastrointestinal parasites [23]. However, in their study that screened wild grasscutter and antelope bushmeat, Abara et al. [24] could not detect *Toxocara* spp. [24]. This difference may be attributable to the techniques. In this study, *Toxocara* sp. was detected after a helminthological autopsy. Abara et al. [24] applied only the concentration technique (formalin ether method) to recover ova and larva from fecal samples.

In this study, the least prevalent genera were *Taenia* spp., detected only in the one examined genet (infection rate of 0.9%). Okoye et al. [19] also detected *Taenia* species only in one animal species (*O. cuniculus*) among all that they examined [19]. Abara et al. [24] detected *Taenia* sp. in the two animal species they screened. Nevertheless, Omonona et al. [23] could not detect *Taenia* spp. in their study [23]. This difference may be due to the geographical and ecological characteristics of the study area and the types of bushmeat screened. In the present study, *Taenia* spp. was detected in carnivorous species, a genet known for its appetite for small mammals such as small rodents. However, small rodents are described as natural intermediate hosts of the cestodes and contribute to the spread of taeniid eggs [25]. In addition, taeniid eggs might survive up to eight months under suitable conditions and can be spread by shoes, animal paws, flies, or other vectors in the endemic area [26]. It may explain why Okoye et al. [19] reported that only a wild rabbit was infected since it is known as a burrower animal.

Among the 15 parasite taxa recorded, *Strongylids* species, *Strongyloides* spp., *Trichuris* spp., and *Ascaris* spp. were detected in the most commonly eaten game animals antelopes, duiker, Cercopithecus (small monkeys) and porcupines. Although in lower proportions, *Entamoeba* spp. was also detected in the same animal species. These parasitic genera comprise pathogenic species for humans. In addition to the presence of potential human pathogenic parasites in antelopes, duiker, Cercopithecus (small monkeys), and porcupines, the highest infestation rates were reported in these animals. In their study in Cameroon, Pourrut et al. detected a prevalence of gastrointestinal parasitism of 92% in wild and captive monkeys [27]. In addition, wild monkeys were reported to have a higher helminth infection rate than pet monkeys. Thus, the consumption of offal from these animals could constitute a risk for consumers. In their work, Okoye et al. revealed that wild animals are heavily infested with parasites that are potentially pathogenic to humans. Okoye et al. [19] and Sowemimo and Asaolu [28] hypothesize that human contamination is possible through hunting dogs nourished by the hunters with raw offal of wild animals [19,28], because this practice could constitute an appropriate route for transmitting zoonotic parasites to humans, as dogs roam the streets unsupervised in many tropical communities, defecate and contaminate the environment with helminth eggs and larvae, cysts of protozoa and other infectious agents. In addition, Pourrut et al. [27] stated that consuming meat or infested viscera is the most frequently observed means of transmission.

Moreover, parasites, as potential pathogens, constitute a burden to wildlife and domestic animal because of the physiological distress and mechanical injuries they cause to animals [29]. The work of Graber et al. [30] in Chad and the Central African Republic on the helminths of some wild artiodactyls belonging to the Bovidae and Suidae families showed that the trematodes *Fasciola gigantica* and *Paramphistomum* sp. are specific parasites of ruminant bovids such as antelope and duiker. These results corroborate this study in which antelopes and duiker were the only animal species infected by the *Fasciola* eggs. *Fasciola gigantica*, or the great liver fluke, is the causative agent of fascioliasis, a disease affecting ruminants. It can be transmitted to humans after consuming livers infected with liver flukes.

The lack of difference in prevalence related to age is common. Okoye et al. [19] showed that the age category (adult vs. young) had no significant difference in the prevalence of endoparasites in wild animals in the ecological zone of Nsukka. Apio et al. [31] reported a similar tendency in the bushbuck *Tragelaphus scriptus* from the Queen Elizabeth National Park, Uganda. It can be attributed to adults and juveniles living together in the same ecological area, with a great chance of sharing many things, such as parasitic infection.

Overall, the sex difference in parasitism was not observed in our studied animal species’ prevalence of gastrointestinal parasites. However, a significant difference was observed for *Paramphistomum* spp. This result agrees with Okoye et al. [19], reporting that differences related to sex are expected, with higher parasitism mainly observed in the male of many animal species [19]. According to [31], the sex-related differences are attributed to male hormones that weaken immune functions, favoring parasites’ growth and success in their gut.

## 5. Conclusions and Limitations

It is concluded that the most consumed bushmeat in the Department of Zadie is host of various gut parasites taxa with some parasitic agents infecting humans and their animals. Therefore, the risk of contracting zoonoses for humans is high if proper precautions are not taken while manipulating those animals. The identification of the species was not possible in this study and will require further investigation using different techniques, such as stool culture and molecular analysis. As the parasite load could not be determined for each parasite because of the method of preservation of the samples, it would require working on fresh samples. In addition, this study should be extended to other regions and carried out on a much larger and more representative sample of game species consumed in the country.

## Figures and Tables

**Table 1:** Overall data on the studied animals.

Village	N°	Sex		Age		Type of Bushmeat							
		Males	Females	Young	Adult	Antelope	Duiker	Porcupine	Small Monkey	Pangolin	Nandinia	Genet	Crocodile
Ekata	10	5	5	0	10	1	8	1	0	0	0	0	0
Ilahounéné	1	0	1	0	1	0	1	0	0	0	0	0	0
Mékouma	12	7	5	3	9	3	8	1	0	0	0	0	0
Malassa	1	0	1	0	1	1	0	0	0	0	0	0	0
Ntolo	5	3	2	0	5	2	0	2	1	0	0	0	0
Ego-Poma	4	3	1	0	4	0	3	1	0	0	0	0	0
Grand-Etoumbi	34	15	19	7	27	6	17	3	5	1	1	0	1
Zoula	9	5	4	3	6	0	5	4	0	0	0	0	0
Etchéla-Edounga	21	7	14	3	18	6	11	3	0	0	1	0	0
Komambela	13	8	5	1	12	2	5	3	2	0	0	1	0
Malouma	3	1	2	0	3	3	0	0	0	0	0	0	0
Total	113	54	59	17	96	24	58	18	8	1	2	1	1

**Table 2:** Parasitic form and infestation rates of identified parasite genera.

Parasite Diversity	Adult or Larvae	Eggs or Cyst	Infestation Rate (%)
*Strongylids* eggs	√	√	54%
*Ascaris* spp.	√	√	18.6
*Balantidium* spp.	-	√	10.6
*Capillaria* spp.	-	√	7.96
*Eimeria* spp.	-	√	15.04
*Entamoeba* spp.	-	√	7.96
*Enterobius* spp.	-	√	7.08
*Fasciola* spp.	-	√	15.9
*Mammomonogamus* spp.	-	√	4.42
*Paramphistomum* spp.	-	√	18.6
*Protostrongylus* spp.	√	-	4.42
*Strongyloïdes* spp.	-	√	15.6
*Taenia* spp.	√	-	0.9
*Toxocara* spp.	√	-	6.2
*Trichuris* spp.	√	√	34.51

**Table 3:** Parasite infestation rates in percentage by animal species.

Parasites Taxa		Animal Species							
		Antelope	Nandinia	Crocodile	Duiker	Genet	Pangolin	Cercopithecus	Porcupine
		(*n* = 24)	(*n* = 2)	(*n* = 1)	(*n* = 58)	(*n* = 1)	(*n* = 1)	(*n* = 8)	(*n* = 18)
*Strongylid* species	Eggs	**75**	**0**	**0**	**41.4**	**100**	**100**	**50**	**72.2**
	Adult	4.2	0	0	0	0	0	0	0
	Total	75	0	0	41.4	100	100	50	72.2
*Strongyloïdes* spp.	Eggs	**20.8**	**0**	**0**	**7**	**100**	**0**	**25**	**50**
	Adult	0	0	0	0	100	0	0	5.5
	Total	20.8	0	0	7	100	0	25	50
*Eimeria* spp.	Oocysts	20.8	0	0	17.5	0	0	0	11.1
*Balantidium* spp.	Oocysts	8.3	0	0	8.8	100	0	0	22.2
*Entamoeba* spp.	Oocysts	16.7	0	0	3.5	0	0	25	5.6
*Ascaris* spp.	Eggs	20.8	50	0	19.0	0	0	0	11.1
	Adult	0	0	0	5.2	0	0	0	0
	Total	20.8	50	0	**20.7**	0	0	0	11.1
*Trichuris* spp.	Eggs	42	50	0	26	100	0	87.5	28
	Adult	17	50	0	7	0	0	37.5	33.3
	Total	42	50	0	**26**	100	0	**87.5**	**39**
*Capillaria* spp.	Eggs	0	0	0	3.5	0	100	25	22.2
*Protostrongylus* spp.	Larvae	8.3	0	0	0	0	0	12.5	11.1
*Enterobius* spp.	Eggs	0	0	0	5.3	0	0	25	16.7
*Fasciola* spp.	Eggs	37.5	0	0	15.8	0	0	0	0
*Paramphistomum* spp.	Eggs	25	0	0	26.3	0	0	0	0
*Mammomonogamus* spp.	Eggs	12.5	0	0	1.8	0	0	0	5.6
*Taenia* spp.	Adults	0	0	0	0	100	0	0	0
*Toxocara* spp.	Adults	4.2	0	0	5.2	0	0	37.5	0

**Table 4:** Distribution and prevalence of parasite species reported to the gender.

Distribution by Gender	Males		Females		χ^2^	*df*	Comparison Test	S	M+	F+
	N/53	P (%)	N/59	P (%)			*p*-value			
*Strongylids* species	**29**	**54.7**	**31**	**52.5**	**0.92**	**2**	**0.63**	**−**	**+**	
*Strongyloïdes* spp.	**7**	**13.2**	**14**	**23.7**	**2.02**	**1**	**0.15**	**−**		**+**
*Eimeria* spp.	**10**	**18.9**	**7**	**11.9**	**1.06**	**1**	**0.30**	**−**	**+**	
*Balantidium* spp.	**5**	**9.4**	**7**	**11.9**	**0.12**	**1**	**0.72**	**−**		**+**
*Entamoeba* spp.	**3**	**5.7**	**6**	**10.2**	**-**	**1**	**0.49**	**−**		**+**
*Ascaris* spp.	**8**	**15.1**	**13**	**22.0**	**0.88**	**1**	**0.34**	**−**		**+**
*Trichuris* spp.	**18**	**33.9**	**20**	**33.9**	**5.1**	**1**	**0.99**	**−**		
*Capillaria* spp.	**2**	**3.8**	**7**	**11.9**	**-**	**1**	**0.17**	**−**		**+**
*Protostrongylus* spp.	**0**	**0**	**4**	**6.8**	**-**	**1**	0.12	**−**		**+**
*Enterobius* spp.	**2**	**3.8**	**5**	**8.5**	**-**	**1**	0.43	**−**		**+**
*Fasciola* spp.	**9**	**17.0**	**11**	**18.6**	**-**	**1**	0.81	**−**		**+**
*Paramphistomum* spp.	**10**	**19.0**	**3**	**5.1**	**-**	**1**	0.03	**+**	**+**	
*Mammomonogamus* spp.	2	3.8	**3**	5.1	-	1	1	**−**		**+**
*Toxocara* spp.	4	7.5	**0**	0	-	1			**+**	
*Taenia* spp.	1	1.9	**0**	0	-	1			**+**	
									**5**	**9**
**Mean**	**7.9**	**14.9**	**10.2**	**17.3**						
**Median**	**7.0**	**13.2**	**7.0**	**11.9**						

N = number of positive specimens; P = prevalence; S = + test significant; + higher prevalence in males (M) or females (F).

**Table 5:** Distribution and prevalence of parasite species reported to the age of different hosts collected during the study period.

Distribution by Age	Adult		Young		*χ* ^2^	*df*	Comparison Test	S	A+	Y+
	N/96	P (%)	N/17	P (%)			*p*-value			
*Strongylids* species	52	54.2	9	53		1	1	−		+
*Strongyloides* spp.	19	19.8	2	11.8		1	0.66	−	+	
*Eimeria* spp.	14	14.6	3	18.7		1	1	−		+
*Balantidium* spp.	10	10.4	2	11.8		1	1	−		+
*Entamoeba* spp.	9	9.4	0	0		1	0.41	−	+	
*Ascaris* spp.	17	17.7	4	23.5		1	0.82	−		+
*Trichuris* ssp.	35	36.5	4	23.5		1	0.45	−	+	
*Capillaria* spp.	6	6.25	3	17.65		1	0.27	−		+
*Protostrongylus* spp.	2	2.1	3	17.65		1	**0.025**	+		+
*Enterobius* spp.	7	7.3	1	5.88		1	1	−	+	
*Fasciola* spp.	15	15.6	3	17.6		1	1	−		+
*Paramphistomum* spp.	17	17.7	4	25		1	0.73	−		+
*Mammomonogamus* spp.	5	5.2	0	0		1	1	−	+	
*Toxocara* spp.	5	5.3	2	13.33		1	0.54	−		+
*Taenia* spp.	1	1.04	0	0		1	1	−	+	
									6	9
Mean	15.5	16.1	2.7	16.8						
Median	14	14.6	3	18.5						

N = number of positive specimens; P = prevalence; S = + test significant; + higher prevalence adults (A) or young (Y).

## Data Availability

The data presented in this study are available on request from the corresponding author.
